# Screening families of patients with premature coronary heart disease to identify avoidable cardiovascular risk: a cross-sectional study of family members and a general population comparison group

**DOI:** 10.1186/1756-0500-3-132

**Published:** 2010-05-11

**Authors:** Helen J Thompson, Alastair CH Pell, Judith Anderson, Clara K Chow, Jill P Pell

**Affiliations:** 1 Section of Public Health, University of Glasgow, Glasgow, G12 8RZ, UK; 2 Department of Cardiology, Monklands Hospital, Airdrie, ML6 0JS, UK; 3 Population Health Research Institute, McMaster University, Hamilton, L8L 2XZ, Canada

## Abstract

**Background:**

Primary prevention should be targeted at individuals with high global cardiovascular risk, but research is lacking on how best to identify such individuals in the general population. Family history is a good proxy measure of global risk and may provide an efficient mechanism for identifying high risk individuals. The aim was to test the feasibility of using patients with premature cardiovascular disease to recruit family members as a means of identifying and screening high-risk individuals.

**Findings:**

We recruited family members of 50 patients attending a cardiology clinic for premature coronary heart disease (CHD). We compared their cardiovascular risk with a general population control group, and determined their perception of their risk and current level of screening. 103 (36%) family members attended screening (27 siblings, 48 adult offspring and 28 partners). Five (5%) had prevalent CHD. A significantly higher percentage had an ASSIGN risk score >20% compared with the general population (13% versus 2%, p < 0.001). Only 37% of family members were aware they were at increased risk and only 50% had had their blood pressure and serum cholesterol level checked in the previous three years.

**Conclusions:**

Patients attending hospital for premature CHD provide a mechanism to contact family members and this can identify individuals with a high global risk who are not currently screened.

## Findings

Primary prevention of cardiovascular disease (CVD) is most effective if people are selected for intervention on the basis of their overall cardiovascular risk [1]. The Scottish Intercollegiate Guidelines Network (SIGN) recommends treatment of anyone at more than 20% risk of a cardiovascular event over the subsequent 10 years [1]. In Scotland, this is determined using the ASSIGN risk score derived from data on individual risk factors: age, sex, socioeconomic status, family history of CVD, cigarette smoking, systolic blood pressure, diabetes, and total and HDL cholesterol [2]. However, determining which members of the general population have a high cardiovascular risk score is problematic. In England, the Department of Health has advocated mass screening of the whole population [3], but this is a high cost, low yield strategy [4], and some people are not in regular contact with primary care.

Cardiovascular disease tends to aggregate in families as a result of both genetic predisposition and clustering of adverse lifestyles. In Scotland, the 28% of individuals whose parents die of CVD account for 61% of everyone with a high global cardiovascular risk [4]. Therefore, targeting screening at those with a family history of CVD offers a cost effective alternative to mass screening [4]. Guidelines already advocate screening of people with a family history of CVD [1,5]. General Practitioners are expected to record family history, along with other risk factors, and screen individuals accordingly, but previous surveys suggest that this strategy has not been effective [6]. The aim was to test the feasibility of using patients attending hospital for premature coronary heart disease to identify and contact family members as a means of targeting screening at high-risk individuals. The objectives were to determine current levels of awareness and screening among family members and their uptake of screening.

We recruited 50 consecutive patients attending the cardiology outpatient clinic at Monklands Hospital, Scotland, for premature CHD. Premature CHD was defined as chronic stable angina plus angiographic confirmation of >50% stenosis in a man less than 55 years of age or a woman less than 65 years of age. Patients provided contact details of their family members, defined as siblings, offspring over 20 years of age and co-habiting partners. Patients were excluded from the study if they had no family members

Relatives and partners were then contacted directly to invite them to attend the index patient's hospital for screening. We used structured, nurse-administered questionnaires to collect data on demographic information, lifestyle cardiovascular risk factors and medical history. Participants were asked whether they considered their risk of cardiovascular disease to be the same as the general population or higher and whether they had had their blood pressure and cholesterol level checked over the previous three years. The research nurse recorded anthropometric measurements and resting blood pressure, and took a fasting blood sample. Written feedback on cardiovascular risk factors was provided to participants and their general practitioners. Approval for the study was granted by the Lanarkshire Medical Research Ethics Committee.

The Scottish Health Survey was used to identify a general population comparison group. Subjects with a family history of premature cardiovascular disease were excluded from the comparison group [7]. We used the Scottish Health Survey to identify and randomly select comparison individuals matched to family members (2:1) by age, sex and deprivation quintile. Family members were compared with the general population group in terms of both individual risk factors and ASSIGN score. Continuous variables were compared using paired t and Wilcoxon signed rank tests for parametric and non-parametric data respectively. Categorical data were compared using McNemar's chi-squared and exact tests, and ordinal data were compared using conditional logistic regression. Statistical significance was assumed at the 5% level. All statistical analyses were performed using SPSS v15.0 software.

**Figure 1:**
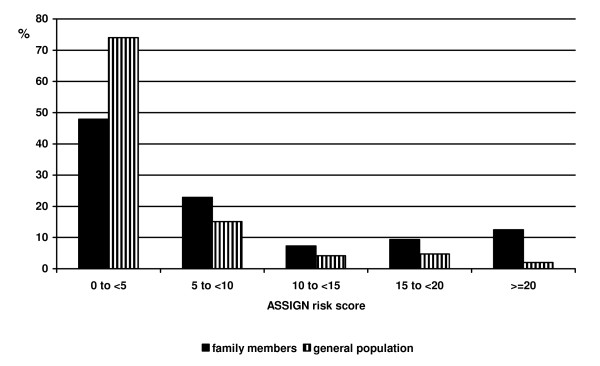
**Distribution of global cardiovascular risk among family members and general population controls**.

The 50 index patients had a median age at diagnosis of 53 years (IQR 49-53), with a median 3 year (IQR 1-7) delay between diagnosis and recruitment to the study. Of the 290 family members listed by patients, 103 (36%) attended for screening; a mean of 2 family members per patient. The 103 family members comprised 27 (26%) siblings, 48 (47%) adult offspring and 28 (27%) partners. Participation rates among siblings, offspring and partners were 16%, 57% and 76% respectively. The most common reason cited for non-participation was living too far from the hospital. Family members had a median age of 41 years and 43% were male. Five (5%) already had established CHD. Compared to members of the general population with no family history, family members were significantly more likely to smoke, be obese, and have hypertension and diabetes (Table 1). ASSIGN risk scores were higher in family members than the general population (median 5.39 versus 2.68, p < 0.001) (Figure 1). Twelve (13%) family members had an estimated probability of a major cardiovascular event in the next 10 years of at least 20% compared with only 4 (2%) people from the general population (p < 0.001). Ninety seven (94%) family members provided information on perceived risk and previous screening. Only 36 (37%) family members perceived their risk of developing CHD in the future to be higher than the general population. Awareness of increased risk was higher among offspring (48%) than siblings (34%), and lowest among partners (19%). Among family members, only 48 (50%) had had both their blood pressure and cholesterol checked within the past three years, and 33 (34%) within the past year.

**Table 1:** Cardiovascular risk factors among family members and general population controls.

	Family members N = 103	General population N = 206	P value*
	**N (%)**	**N (%)**	
**Smoking status**			
Never	40 (39)	118 (57)	< 0.001
Ex	20 (19)	42 (20)	
current	43 (42)	46 (22)	
**Body composition**			
BMI ≥ 25	76 (75)	121 (59)	0.009
WHR >0.8 (F), >0.9 (M)	72 (72)	114 (57)	0.012
Waist >88 cm (F), >102 cm (M)	46 (46)	55 (28)	0.001
**Hypertension**	35 (35)	50 (25)	0.071
**Diabetes**	9 (9)	0 (0)	< 0.001
**Cholesterol:HDL ratio >4.0**	52 (51)	64 (31)	0.001
			
**Established CHD**	5 (5%)	0 (0%)	0.004
			
	**Median (IQR)**	**Median (IQR)**	
**Number of cigarettes smoked**	15 (10, 20)	12 (6, 16)	0.049
**Systolic blood pressure**	129 (119, 142)	124 (115, 133)	0.005
**BMI**	28 (25, 32)	26 (24, 28)	< 0.001
**HDL cholesterol**	1.2 (1.0, 1.6)	1.5 (1.2, 1.8)	< 0.001
**Waist (cm)**	94.5 (86.1, 104.0)	87.2 (81.2, 97.0)	< 0.001
			
	**Mean (SD)**	**Mean (SD)**	
**WHR**	0.89 (0.09)	0.86 (0.07)	< 0.001
**Total cholesterol**	5.3 (1.3)	5.4 (1.0)	0.341

N number, BMI body mass index, WHR waist hip ratio, cm centimetres, F female, M male, HDL high density lipoprotein, CHD coronary heart disease

*paired t test for WHR and total cholesterol, Wilcoxon paired signed rank test for remainder of continuous variables, Conditional logistic regression for smoking status, McNemar's exact test for diabetes and established CHD, McNemar's χ^2 ^test for remainder of categorical variables

Our study demonstrated the feasibility of using patients with premature CHD as a means by which to identify and contact people with high global risk who are not currently screened. Consistent with previous studies, our results demonstrated that the first degree relatives and partners of patients with premature CHD had an increased prevalence of individual risk factors and a higher overall risk of future coronary events. In spite of existing guidelines recommending screening of people with a family history, family members demonstrated a low awareness of their increased risk and a lack of screening.

Received wisdom suggests that primary prevention should be targeted at individuals with a high global cardiovascular risk rather than on the basis of individual risk factors [1]. Research has focused on developing risk prediction models and identifying clinical and cost effective intervention strategies, such as lipid-lowering therapies. By contrast, there has been a paucity of research into how best to identify which members of the general population have a high global risk. In the absence of evidence to the contrary, policy-makers have promoted mass screening as the preferred strategy [3]. However, such a strategy is difficult to deliver in practice and the absolute cost is high. Targeting high risk sub-groups of the population such as individuals in deprived communities and those with a family history offers the potential to identify more than 84% of those at high global risk by screening only 41% of the population [4]. The merits of mass screening are questionable since a further 59 people would need be screened to identify an additional high risk individual at a cost of £1,358 [4].

Published studies have consistently demonstrated that people with a family history of premature CHD have a significantly increased risk of developing CHD, due to a combination of shared genetic predisposition and shared lifestyle [8-10]. Guidelines already exist recommending screening of people with a family history [1,5], yet less than half of the family members in our study had had their blood pressure and cholesterol measured in the past three years. In the EuroAspire II Survey even fewer (11.1% of siblings and 5.6% of offspring) had undergone screening specifically "as a result of CHD in their family" [6]. This may be due, in part, to lack of awareness that risk can cluster in families. In previous studies, around half of people with a parental history of premature CHD were aware they were at increased risk [11,12]. In our study the figures were even lower, with only 41% of first degree relatives aware of their increased risk. Partners of patients with CHD have also been demonstrated to be at increased risk, as a result of shared lifestyle [13]. Despite this partners have largely been excluded from research studies and guidelines [1,5], and, in our study, less than one-fifth of partners were aware that they were at increased risk.

The strengths of our study included recruitment of unselected patients and inclusion of partners, as well as first degree relatives. Previous studies have tended to compare individual risk factors, whereas our study also compared global risk. Our comparison group was matched at an individual level for age, sex and deprivation quintile and was drawn from the same population. They were not recruited as part of the same study but the same questions and measurements were administered. A limitation of this study, as with previous studies, is the exclusion of fatal index cases of CHD. Compared with non-fatal cases, family members of fatal cases may have a different risk profile, risk perception and level of motivation.

Our patients were recruited a median of three years after diagnosis, during which time some family members may have been screened and treated. Therefore, our results are likely to underestimate the level of increased risk among family members at the time of diagnosis. The fact that more than half of family members had not been screened since diagnosis demonstrates that existing strategies are not effective. We believe that the failure of existing guidelines has resulted from a reliance on general practitioners recording family history as a risk factor and targeting individuals accordingly. An alternative approach is to ask patients presenting with premature CHD to provide details of their family members. Compared with screening of the general population, recruitment triggered by a diagnosis of CHD in a family member could lead to greater motivation to attend screening and modify lifestyle. This approach is routine practice for a number of familial cancers. In cardiological practice it is used for familial dyslipidaemia but, as yet, not for general cardiovascular screening.

In our study, only 36% of invited family members attended. We can only speculate as to whether attendees were representative of all family members and, therefore, whether the results are generalisable. Recruitment bias could potentially operate in either direction with participation being more likely either among those with pre-existing awareness and concerns, or those hitherto neglected. In our study, recruitment and screening were both based in a single hospital. We did not have ethics committee permission to question non-participants as to their reason for non-attendance. However, excessive distance from the clinic was commonly volunteered as a reason for non-attendance. Extension of the screening service to include multiple sites across the country, would probably improve uptake.

Our findings suggest that patients presenting with premature CHD may provide a mechanism to identify family members and thereby improve cardiovascular screening. This strategy should be evaluated in a larger, multi-centre study.

## List of abbreviations

ASSIGN: assessing cardiovascular risk using SIGN guidelines; CHD: coronary heart disease; CVD: cardiovascular disease; HDL: high density lipoprotein; MWU: Mann Whitney U; SIGN: Scottish Intercollegiate Guidelines Network.

## Competing interests

The authors declare that they have no competing interests.

## Authors' contributions

ACHP had the original concept. JPP designed the study. CKC designed the questionnaire. MJT and JA recruited the participants and collected the data. JHT analysed the data. JPP drafted the manuscript. All authors contributed to interpretation of the data, redrafting of the manuscript and approved the final version. JPP is the guarantor.
